# Opioid-related polysubstance use and its effect on mortality and health resource utilization among trauma patients

**DOI:** 10.1186/s40621-023-00459-0

**Published:** 2023-10-23

**Authors:** Safalta Khadka, James M. Bardes, Mohammad A. Al-Mamun

**Affiliations:** 1 Department of Pharmaceutical Systems and Policy, School of Pharmacy, West Virginia University, Morgantown, WV 26505 USA; 2 Division of Trauma, Acute Care Surgery and Critical Care, Department of Surgery, West Virginia University, Morgantown, WV 26505 USA

**Keywords:** Opioid, Polysubstance, Pre-injury substance use, Trauma, Outcomes

## Abstract

**Background:**

Pre-injury opioid use is common, but the effects of opioid-related polysubstance use on mortality and health resources utilization (HRU) have not been investigated yet. The objective of this study was to investigate the effects of opioid-related polysubstance use on mortality and HRU among patients in trauma centres in the US.

**Methods:**

We conducted a retrospective cross-sectional study using the US National Trauma Databank from the year 2017 to 2019. Patients (≥ 18 years of age) who tested positive for opioids were included. Patients were analysed based on the number of substances used (i.e., opioids only, two substances (opioids + 1 substance), and three or more than three substances (opioids +  ≥ 2 substances)), and polysubstance by type (i.e., opioids only, opioids and alcohol, opioids and stimulants, opioids and benzodiazepine, and other combinations). Multivariate logistic regression was used to determine the association between polysubstance use, mortality and HRU (i.e., need for hospital admission, ICU, and mechanical ventilation).

**Results:**

Both polysubstance by number and type analyses showed that opioid-related polysubstance use was not significantly associated with mortality compared to opioids only. The odds of hospital admission were higher among the opioids and benzodiazepines group (OR 1.15, 95% CI 1.06–1.24, p < 0.01). The need for ICU was magnified using benzodiazepines and stimulants with opioids (OR 1.44, 95% CI 1.27–1.63, p < 0.01) when compared to the opioids only group.

**Conclusion:**

Opioid-related pre-injury polysubstance use was associated with higher HRU in trauma patients. The evidence can be used by policymakers and practitioners to improve patient outcomes in trauma centers.

**Supplementary Information:**

The online version contains supplementary material available at 10.1186/s40621-023-00459-0.

## Introduction

Substance use is prominent among patients admitted to trauma centers in the US. It is estimated that approximately 40–50% of trauma patients are using multiple substances before their injury (Cornwell et al. 1998). Additionally, 50–70% of the patients tested positive for illegal substances or alcohol during hospital admission in trauma centres (Demetriades et al. 2004). Polysubstance use—defined as concurrent or sequential consumption of two or more substances within a short period of time. It is also very common among people who use opioids (Hassan and Foll 2019) and is a contributing factor to traumatic injuries (Cowperthwaite and Burnett 2011). For instance, a motor vehicle driver with polysubstance use has higher odds of getting into traffic accidents than those abusing a single substance (Dubois et al. 2015).

Nearly 63% of opioid overdose deaths involved one or more additional substances like cocaine, methamphetamine, or benzodiazepine (Gladden et al. 2019). Depressants (e.g., alcohol and benzodiazepine) and stimulants (e.g., amphetamine and methamphetamine) are the most used substances with opioids (NIDA 2022). Among 106,000 deaths due to overdose in the US in 2021, 67% of them involved an opioid (NDA 2022). Deaths involving polysubstance use with opioids are common, making polysubstance use a major problem among opioid users (Compton et al. 2021; Barocas et al. 2019).

According to several pharmacoepidemiologic studies, patients who are on benzodiazepine prescriptions and concurrently use opioid analgesics pose a higher risk of death from drug overdose than those simply receiving opioids only (Park et al. 2015; Yang et al. 2020; Hernandez et al. 2018; Gibbons et al. 2021). Similarly, when opioids and alcohol are consumed together, there are more health hazards than when consumed alone (Witkiewitz and Vowles 2018). Alcohol and opioids have unfavourable pharmacodynamic interactions, such as an increased risk of respiratory depression and overdose-related deaths (White and Irvine 1999). Methamphetamine and opiate use are associated with hepatitis C virus, severe mental illness, and increased use of injectable drugs (Korthuis et al. 2022 Aug). As a result, the co-use of substances increases the risk of fatal overdose significantly among patients who consume opioids.

Drug screening during the admission in trauma centres is the focal point of information gathering for substance use. Many studies used drug screening results to examine the effect of pre-injury substance use on the different clinical outcomes of trauma patients (Cowperthwaite and Burnett 2011; Andelic et al. 2010; Karnick et al. 2021; Cheng et al. 2016; Yeung et al. 2013; Culhane and Freeman 2020). As pre-injury substance use is one of the major contributors to traumatic injury, its effect on different health outcomes (e.g., mortality and cardiac complications) were studied previously (Cheng et al. 2016; Culhane and Freeman 2020; Cannon et al. 2014). Among them, several studies have shown that pre-injury opioid use was not associated with mortality among trauma patients but was instead linked with an increased length of hospital stay (Cheng et al. 2016; Pandya et al. 2015). As polysubstance use with opioids has increased recently (BJA.gov 2018), it is essential to explore the effect of opioid-related polysubstance on outcomes among trauma patients (cinc. 2020).

Health resource utilization (HRU) is an important measuring parameter to ensure the best possible health outcomes for patients while saving a substantial economic burden in the trauma centres (Ungar et al. 1998). Notably, use of ICU and mechanical ventilation are the most critical resources in the hospital setting. They are not only associated with patient’s outcome and health care system’s economic landscape but also are equally significant from the payer’s perspective. Additionally, within the realm of pre-injury polysubstance use, identifying the association between HRU for each type of pre-injury substance use holds the promise of enhancing decision-making processes both at the patient and health care system levels. For instance, gaining insights of the higher odds of ventilator use associated with polysubstance use can inform more precise and efficient resource allocation strategies. These insights have the potential to optimize health care delivery, improve patient outcomes, and alleviate the strain of economic burden faced by health care system.

To date, the role of opioid-related polysubstance use on trauma outcomes such as mortality and HRU has not been documented. Therefore, this study aimed to determine the effect of pre-injury opioid-related polysubstance on mortality and HRU among trauma patients. We hypothesized that pre-injury opioid-related polysubstance use is associated with an increased risk of mortality and increased HRU when compared to the use of opioids only.

## Materials and methods

### Study design and population

**Fig. 1:**
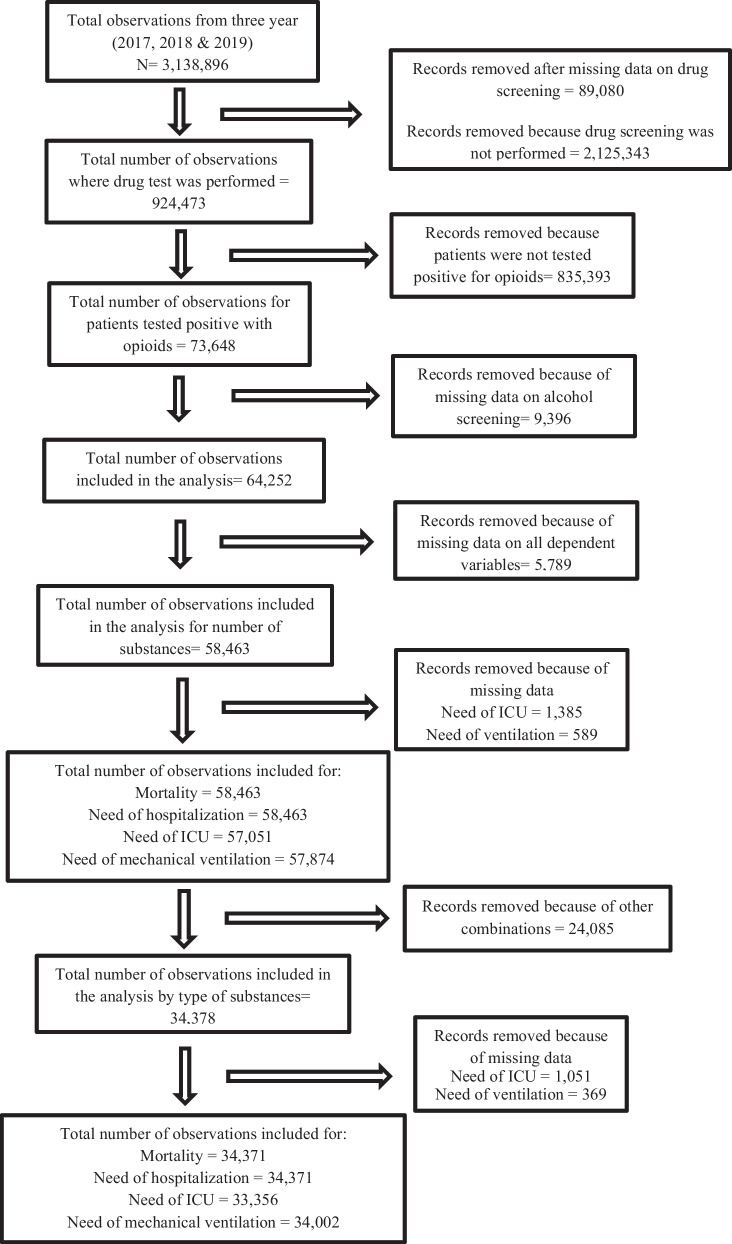
Flow chart of patients included in the analysis

We conducted a retrospective cross-sectional study using a large national database of trauma centers, the National Trauma Data Bank (NTDB), from the year 2017 to 2019. The databank was obtained from the American College of Surgeons (ACS) and further approved by the Institutional Review Board of West Virginia University (IRB: 2201506253). All the patients included in the study were based on their unique admission records. It includes information on the sociodemographic, injury, emergency department, hospital procedure, diagnosis, hospital complications, and care process measures for patients. The NTDB consists of urine drug screening information for 12 different types of drugs, as well as blood alcohol levels. The drug screening variable was defined as the first recorded positive drug screen results within 24 h after the first hospital encounter and is presented as a binary variable in the database. The other variable “drug screening tested” provided the information whether the screening was performed or not along with missing data.” The drugs included in the screening were opioids, amphetamine, barbiturates, benzodiazepine, cocaine, methamphetamine, ecstasy, methadone, oxycodone (coded as an opioid in this study), phencyclidine, tricyclidine antidepressants, cannabinoids, and others. In this study, amphetamine, cocaine, methamphetamine, and ecstasy were grouped as stimulants, and patients with Blood Alcohol Concentration (BAC) > 0.08 were termed as alcohol positive. The missing values (9396) for blood alcohol concentration were excluded from the study (Fig. 1).

Trauma patients that were ≥ 18 years of age who tested positive for opioid use were included in this study. They were analyzed based on polysubstance use by number and by type of substances. The comparator group of the study is the group of opioids only. Polysubstance by number were categorized into groups of those who used opioids only, two substances: opioids + one substance, and polysubstance or three or more substances: opioids +  ≥ 2 substances. Polysubstance by type was categorized by either opioid only or opioids with one of the following: alcohol, stimulants, or benzodiazepine. These combinations were selected based on a descriptive analysis of the frequently observed combination of substances positive with opioids. Other polysubstance combinations by substances were excluded in this study.

### Variable selection

The primary and secondary outcomes were mortality and HRU, respectively. The HRU included need(s) for hospital admission, intensive care unit (ICU) admission, and mechanical ventilation.

The covariates that were included in our analysis were age, sex, race, payment method, hypertension, diabetes, smoker, congestive heart failure (CHF), chronic renal disease (CRD), alcohol use disorder (AUD), and substance use disorder. Substance use disorder and drug use disorder variables were combined into a single variable named substance use disorder (SUD) to make the data uniform for 3 years. Following the NTDB guidelines, we also considered hypotension, pulse rate, systolic blood pressure (SBP), total Glasgow Coma Scale (GCS), mechanism of injury, and injury severity score (ISS), as covariates in investigating the odds of mortality (Hashmi et al. 2018). We used the same covariates in examining the odds of hospital admission, ICU admission, and need for mechanical ventilation.

### Data analytic strategy

#### Missing data

Missing value for dependent variables (i.e., mortality, hospital admission, ICU admission, and need for mechanical ventilation) and independent variables (substance use) were removed. We then implemented a mean imputation technique for MAR values for three covariates i.e., total GCS, SBP, and pulse rate using *MICE* package in R for missing values (Zhang 2016). For other categorical covariates such as age, sex, primary method of payment, race, trauma type, mechanism of injury and injury severity, missing values were labelled as “missing” and were included in the analysis.

#### Statistical analyses

We conducted a descriptive analysis to determine opioid-related pre-injury polysubstance use among trauma patients. Comparisons within the groups based on polysubstance by number and type were done using Chi-square tests for categorical data and an ANOVA test for continuous variables. Bonferroni adjustment was made for multiple comparisons. Furthermore, we conducted Wilcoxon rank test to determine the association between the independent variables and outcome variables (hospital length of stay, ICU days, and ventilator days). The odds ratio (OR) was calculated to estimate the effect of these variables on the primary and secondary outcomes.

A multivariate logistic regression model was used to determine the association of pre-injury polysubstance use with mortality, hospital admission, ICU admission, and the need for mechanical ventilation. The regression model was adjusted for age, gender, race, mechanism of injury, injury severity score, SBP, pulse rate, GCS, AUD, SUD, CHF, CRD, smoker, hypertension, and diabetes. All the statistical analyses were performed using SAS version 9.4. Furthermore, we also explored the effect of polysubstance on mortality and HRU by using patients with no substance as comparison group.

#### Significance

All statistical tests were performed at α value 0.95, and the p-value was termed as significant if the value was ≤ 0.05. To demonstrate the relative effects of polysubstance use on mortality and HRU, an OR with a 95% confidence interval was reported.

## Results

Of 3,138,896 patients, only 924,473 (29.4%) patients were screened for substance use and 89,080 (2.83%) observations were missing for the drug screening variable. Among them, 73,648 (8%) patients were tested positive for opioids. Overall, 58,463 patients met the inclusion criteria. Among them, a total of 17,930 (31%) patients had opioid use only, 20,165 (35%) patients tested positive for two substances, and 20,376 (34.8%) had tested positive for three or more substances. Among the categories of polysubstance by type, 3740 (5%) patients were identified as positive for opioids and alcohol, 4076 (7%) patients for opioids and benzodiazepine, and 6126 (11%) patients for opioids and stimulants. Additionally, 895 (1%) patients tested positive for the combination of opioids, alcohol, and benzodiazepine, and 1611 (3%) patients tested positive for the combination of opioids, stimulants, and benzodiazepine. There were only 34,378 patients who were included in the analysis by substance type. Other combinations were not incorporated for the analysis of type of substances.

### Baseline patient characteristics

Table 1 presents the baseline demographic characteristics of trauma patients for pre-injury polysubstance use by the number of substances. For the age group ≤ 21 years, use of two substances was more common whereas for the older population (i.e., ≥ 65 years), use of opioid only was frequent (60.1%). Age groups 22–34 and 35–44 years, intake of three or more substances were higher than two substances use and opioid only. Interestingly, greater percentage of males (37.2%) tested positive for three or more substances and opioid + 1 substance compared to females. When comparing race among the groups, African Americans tested positive more often for two substances and three or more substances use compared to opioids only. Additionally, Pacific Islander also had higher intake of additional substances with opioids. Uninsured patients had greater use of three or more substances, followed by two substances and then opioids only (44% vs. 35% vs. 20%, p < 0.001). Additionally, patients with a history of AUD, SUD, and smoking had significantly (p < 0.001) higher intakes of three or more substances compared to two substances use (AUD: 46% vs. 38% vs. 15%, SUD: 52% vs. 37% vs. 10%, smoker: 22% vs. 39 vs. 45%, respectively). The median GCS was 15, and median SBP was 133 mm hg among patients tested for three or more substances.

**Table 1:** Demographics of trauma patients with pre-injury polysubstance use by number of substances

Characteristics	Opioids	Opioids + 1 substance	Opioids + ≥ 2 substance	p-value
Age yrs, n(%)				< 0.0001
≤ 21	736 (23.6)	1232 (39.6)	1142 (36.7)	
22–34	3216 (19.2)	6115 (36.6)	7362 (44.1)	
35–44	2238 (21.9)	3691 (36.23)	4258 (41.8)	
45–64	5932 (31.1)	6829 (36.2)	6064 (32.21)	
≥ 65	5808 (60.15)	2298 (23.80)	1550 (16.05)	
Sex, n (%)				< 0.0001
Female	6903 (37.9)	5927 (32.5)	5378 (29.5)	
Male	11,026 (27.39)	14,237 (35.36)	14,996 (37.25)	
Missing	1 (25.0)	1 (25)	2 (50.0)	
Race/ethnicity, n (%)				< 0.0001
White	13,587 (32.59)	13,941 (33.4)	14,161 (33.97)	
African American	2214 (21.77)	4053 (39.85)	3903 (38.38)	
Asian	362 (56.83)	154 (24.18)	121 (19.00)	
American indian	120 (18.21)	222 (33.6)	317 (48.10)	
Pacific Islander	21 (21.00)	40 (40.0)	39 (39.00)	
Other	1544 (31.63)	1638 (33.55)	1700 (34.8)	
Missing	82 (24.55)	117 (35.03)	135 (40.42)	
Mechanism Category, n (%)				< 0.0001
Auto versus Pedesterian	632 (24.28)	939 (36.07)	1032 (39.65)	
Fall	6811 (42.08)	5181 (32.01)	4194 (25.91)	
Motorcycle collision	1232 (32.75)	1343 (35.70)	1187 (31.55)	
Motor vehicle collision	5417 (30.27)	6209 (34.69)	6271 (35.04)	
Missing	210 (23.73)	353 (39.89)	322 (36.38)	
Other	2401 (25.76)	3177 (34.08)	3744 (40.16)	
Penetrating	1227 (15.70)	2963 (37.91)	3626 (46.39)	
Payment, n (%)				< 0.0001
Public	8235 (30.50)	9214 (34.14)	9537 (35.34)	
Private	6924 (34.66)	6775 (33.91)	6279 (31.43)	
Uninsured	1664 (20.00)	2986 (35.89)	3670 (44.11)	
Other	954 (37.14)	949 (36.94)	666 (25.92)	
Missing	153 (24.76)	241 (39.00)	224 (36.25)	
Comorbidities n (%)				
Alcohol Use Disorder	824 (15.89)	1973 (38.04)	2390 (46.08)	< 0.0001
Substance Use Disorder	1174 (10.39)	4239 (37.43)	5911 (52.20)	< 0.0001
Cognitive heart failure	744 (51.74)	407 (28.30)	287 (19.96)	< 0.0001
Chronic Renal Disease	208 (55.47)	108 (28.80)	59 (15.73)	< 0.0001
Smoker	3745 (18.50)	7633 (37.70)	8869 (43.80)	< 0.0001
Hypertension	6639 (44.46)	4606 (30.85)	3687 (24.69)	< 0.0001
COPD	1512 (37.95)	1332 (33.43)	1140 (28.61)	< 0.0001
Diabetes	2788 (48.89)	1664 (29.18)	1251 (21.94)	< 0.0001
Injury Severity, n (%)				< 0.0001
Less severe	14,711 (31.40)	16,204 (34.89)	15,935 (34.01)	
Severe	3160 (27.43)	3913 (34.09)	4405 (38.38)	
Missing	59 (41.26)	48 (33.57)	36 (25.17)	
GCS, median	15	15	15	< 0.0001
Pulse Rate, median	87	90	92	< 0.0001
SBP, median mmHG	140	135	133	< 0.0001

*ISS* Injury Severity Score, *COPD* Chronic obstructive pulmonary disease, *GCS* Glasgow Coma Scale, *SBP* Systolic Blood Pressure

Chi-square test was conducted for the categorical variables and Anova was used for the continuous variables

Additional file 1: Table S1 presents the demographics for patients based on polysubstance by type of substances. Age groups ranging from 22 to 34 and 45–64 years were more prone to use the different combinations of the substances. Opioids and stimulants are the most common combination for the age group 22–34 years (25%), 35–44 years (27%) and 45–64 years (18%). The uninsured patients were commonly using the combination of opioids and stimulants (27%), followed by opioids and alcohol (14%). Similarly, among the patients with SUD, 45% of the patients were tested positive for the opioids and stimulants.

The median hospital length of stay was similar among the two substances and three or more substances (Table 2); however, the IQR increased with the higher number of polysubstance uses. Based on the number of substances, polysubstance had the least median ventilator days (2, [IQR 2–7]) while the two substances and opioid-only groups had a similar median (3, [IQR 2–7] vs. 3, [IQR 2–7]). For polysubstance by type, the median hospital length of stay was highest among patients who tested positive for the combination of opioids and benzodiazepine (5, [IQR 3–8]) and the combination of opioids, alcohol, and benzodiazepine (5, [IQR 3–8]). The combination of opioids, alcohol, and benzodiazepine had the highest IQR but the lowest median for ventilator days (2, [IQR 1–8]).

**Table 2:** Within group comparison of Health resources utilization among adult trauma patients who use polysubstance by number of substance and by type of substances

	Hospital admission (%)	Hospital length of stay	ICU admission n (%)	ICU days	Mechanical ventilation n (%)	Ventilator days
		Median (IQR)		Median (IQR)		Median (IQR)
Polysubstance use by number of substances						
Opioid only	13,580 (64)	4 (3–6)	6183 (29)	3 (2–5)	1457 (7)	3 (2–7)
Opioids + 1 substance	14,639 (65)	4 (2–6)	7137 (32)	3 (2–5)	2489 (11)	3 (2–7)
Opioids + ≥ 2 substance	15,055 (68)	4 (2–7)	7529 (34)	3 (2–6)	3286 (15)	2 (2–7)
p-value	< 0.001	< 0.001	< 0.001	< 0.001	< 0.001	< 0.001
Polysubstance use by type of substances						
Opioids only	13,580 (64)	4 (3–6)	6183 (29)	3 (2–5)	1457 (7)	3 (2–7)
Opioids and alcohol	2216 (62)	3 (2–6)	1161 (33)	3 (2–5)	372 (10)	3 (2–6)
Opioids and Stimulants	4543 (64)	4 (2–6)	2226 (31)	3 (2–5)	848 (12)	3 (2–6)
Opioids and benzodiazepines	3140 (66)	5 (3–8)	1824 (38)	3 (2–6)	749 (16)	3 (2–8)
Opioids, alcohol and benzodiazepines	554 (64)	5 (3–8)	387 (45)	3 (2–6)	165 (19)	2 (1–8)
Opioids, stimulants, and benzodiazepines	1178 (62)	4 (3–8)	770 (41)	4 (7)	416 (22)	3 (2–7)
p-value	0.0032	< 0.001	< 0.001	< 0.001	< 0.001	< 0.001

Wilcoxon rank test was performed

### Mortality in adult trauma patients using polysubstance with opioids

The odds of mortality for two substances and three or more substances use compared to opioids only were lower (OR 0.94, 95% CI 0.77–1.15, p = 0.58), (OR 0.82, 95% CI 0.67–1.02, p = 0.08) but were not statistically significant (Table 3).

**Table 3:** Odds of mortality in adult trauma patients after adjusting multivariate logistic regression

Risk Factors	OR^a^ (95% CI)	p-value
Polysubstance by number of substances		
Opioids only	Reference	
Opioids + 1 substance	0.94 (0.77–1.15)	0.58
Opioids + ≥ 2 substance	0.82 (0.67–1.02)	0.08
Polysubstance by types of substances		
Opioids only	Reference	
Opioids and alcohol	0.79 (0.55–1.16)	0.24
Opioids and stimulants	1.15 (0.84–1.58)	0.37
Opioids and benzodiazepines	0.79 (0.59–1.06)	0.12
Opioids, stimulants and benzodiazepines	0.67 (0.39–1.16)	0.13
Opioids, alcohol and benzodiazepines	0.48 (0.23–0.98)	0.04

^a^Logistic regression model was adjusted for age, sex, mechanism of injury, injury severity, Glasgow Coma Scale and systolic blood pressure, pulse rate and different comorbid conditions

Similarly, when evaluating mortality among patients with polysubstance use by substance type, the odds of mortality of different combinations were lower compared to opioids only, except for the combination of opioids and stimulants. The combination of opioids and stimulants had the highest odds of mortality (OR 1.15, 95% CI 0.84–1.58, p = 0.37), while the combination of opioids, alcohol, and benzodiazepine had the lowest odds of mortality (OR 0.48, 95% CI 0.23–0.99, p = 0.05); none was statistically significant. However, the combination of opioids, alcohol, and benzodiazepines were on the borderline to be statistically significant.

When the comparison group was changed from patients positive with opioids to patients negative to all substances in urine drug testing, the result showed the odds of mortality decreased by using opioids (OR 0.86 95% CI 0.75–0.79, p = 0.04) (Additional file 1: Table S2). Furthermore, the odds of mortality were lower for patients using three or more than three substances (OR 0.73 95% CI 0.64–0.83, p < 0.0001) (Additional file 1: Table S2). Use of opioids, alcohol and benzodiazepines had the lowest odds of mortality (OR 0.44 95% CI 0.22–0.87, p = 0.02).

### Healthcare resource utilization in trauma patients using opioid-related polysubstance

Among the polysubstance by number, the use of two substances was associated significantly with an increased risk of hospital admission (OR 1.2, 95% CI 1.14–1.25, p < 0.001), Similarly, the use of two substances or polysubstance was also significantly associated with the need of mechanical ventilation (OR 1.42, 95% CI 1.30–1.55, p < 0.001 vs. OR 1.67, 95% CI 1.52–1.84, p < 0.001) compared to the patients who used opioids only (Table 4). However, the need for ICU admission was not statistically significant for both two substances and three or more substances groups.

**Table 4:** Odds of health service utilization in adult trauma patients after adjusting multivariate logistic regression

	Hospital Admission		ICU Admission		Mechanical Ventilation	
	Odds ratio^a^, CI	p-value	Odds ratio^a^, CI	p-value	Odds ratio^a^, CI	p-value
Polysubstance use by number of substances						
Opioids only	Reference		Reference		Reference	
Opioids + 1 substance	0.98 (0.94–1.03)	0.6	1.02 (0.97–1.08)	0.33	1.42 (1.30–1.55)	< 0.001
Opioids + ≥ 2 substance	1.2 (1.14–1.25)	< 0.001	1.003 (0.95–1.05)	0.9	1.67 (1.52–1.84)	< 0.001
Polysubstance Use by type of substances						
Opioids Only	Reference		Reference		Reference	
Opioids and alcohol combination	0.92 (0.85–1.00)	0.06	0.92 (0.84–1.01)	0.09	1.18 (1.01–1.38)	0.03
Opioids and stimulants	0.89 (0.83–0.96)	0.002	1.08 (1.001–1.17)	0.04	1.49 (1.30–1.69)	< 0.001
Opioids and benzodiazepines	1.1 (1.06–1.24)	< 0.001	1.25 (1.15–1.35)	< 0.001	1.78 (1.56–2.03)	< 0.001
Opioids, stimulants and benzodiazepines	0.95 (0.84–1.06)	0.39	1.44 (1.27–1.63)	< 0.001	2.64 (2.21–3.15)	< 0.001
Opioids, alcohol and benzodiazepines	1.14 (0.98–1.33)	0.07	1.34 (1.14–1.57)	< 0.001	1.80 (1.4–2.28)	< 0.001

^a^Logistic regression model was adjusted for age, sex, mechanism of injury, injury severity, Glasgow Coma Scale, systolic blood pressure, pulse rate and different comorbid conditions

The odds of hospital admission were higher among the opioids and benzodiazepine group (OR 1.15, CI 1.06–1.24, p < 0.001). Interestingly, use of opioids and stimulants decreased the odds of hospitalization (OR 0.89, CI 0.83–0.96, p < 0.001). The ICU admission of those using benzodiazepine and stimulants with opioids was magnified significantly (OR 1.44, CI 1.27–1.63, p < 0.001). Additionally, the odds of need for ventilation were approximately three times higher among patients who tested positive for stimulants and benzodiazepine with opioids compared to patients using opioids only (OR 2.64, CI 2.21–3.15, p < 0.001). Overall, using other substances (alcohol, stimulants, and benzodiazepine) with opioids was associated with increased odds of needing mechanical ventilation.

When compared to patients with no substance group, use of opioids and adding additional substances increased odds of hospital admission but decreased the odds of ICU admission and mechanical ventilation. Use of opioids with benzodiazepines in addition to alcohol or stimulants increased the odds of hospital admission, ICU admission and mechanical ventilation (Additional file 1: Table S3).

## Discussion

One of the challenges of the polysubstance use in trauma patients is trying to navigate which combinations affect outcomes, as there are wide range of possible combinations of substances that people can use. Through this cross-sectional study, we have investigated the effect of pre-injury opioid-related polysubstance use on two major outcomes (mortality and HRU) among trauma patients. Our results have illuminated several important findings.

Our study showed that only 29.4% of patients went through drug screening in the trauma centers which corroborates the study by Silver et al. (2023). Additionally, our results showed that opioids were more frequently used in combination with other substances compared to opioids only. The finding highlights the intricate nature of substance use behaviors, emphasizing the need for a more comprehensive approach to address polysubstance use that considers the potential interaction between various substances. Younger patients in the age group 22–34 years and 35–44 years were more likely to engage in polysubstance use when considering polysubstance by number. Similarly, polysubstance use was also common among uninsured patients. These demographic patterns highlight the vulnerability of specific populations to polysubstance use and warrants for the targeted interventions tailored to these under-served populations.

Within the realm of polysubstance combinations, our research uncovered some noteworthy patterns. The combination of opioids and stimulants was more common, followed by the combination of opioids and benzodiazepine. However, an intriguing finding is that none of these combinations were significantly associated with mortality (Table 3). The result challenges the presumption about the association between specific substance combinations such as opioids and alcohol, opioids and benzodiazepines and fatal outcomes, underlining the multifaceted nature of mortality in trauma patients with polysubstance use. Interestingly, even though the results were not statistically significant, the use of polysubstance showed a decrease in odds of mortality which implied the protective effect of these combinations. Specifically, the association of combination of opioids, alcohol and benzodiazepines was in the borderline with mortality (Table 3). This may be due to the various pharmacologic effect of these drugs, or the robust conclusion may not be drawn due to the limited information on the dosage and timing of the use of these drugs.

Surprisingly, the combination of opioids and alcohol were associated with higher mechanical ventilation use and opioids and stimulants were associated with lower odds of hospital admission. The result is intriguing and requires further investigation. The information on the characteristics of patients and their comorbid conditions should be further explored along with the dose and timing of these substances used.

Our study showed the robust association between the opioid-related two substances and polysubstance use with higher HRU. For instance, the combination of benzodiazepine and opioids along with other substances (alcohol or stimulant) had an overall higher HRU. The result highlights the importance of identifying the specific health care challenges faced by patients using these combinations.

Furthermore, when compared to patients with no substance use, polysubstance use had decreased odds of  mortality. The association between use of opioids and mortality was on the borderline. When compared to other studies, our literature search presented variable findings and clinical outcomes related to the combination and use of substances examined in this research. The effect of opioids on mortality has been widely studied in trauma patients, yet several studies have shown no association (Cheng et al. 2016; Cannon et al. 2014; Pandya et al. 2015). Elsewhere, while the effect of opioids with polysubstance on mortality has not been explicitly reported, research has shown that results vary regarding the association between stimulant use and mortality (Hadjizacharia et al. 2009; Neeki et al. 2018). Satish et al. investigated the association of amphetamine and cocaine separately on mortality, showing that cocaine reduced the odds of mortality among trauma patients, while amphetamine did not show any such association (Satish et al. 2021). As highlighted, though, outcomes vary in the literature (Yeung et al. 2013; Satish et al. 2021). Our study stands unique, as it finds no association between the use of opioids and stimulants with mortality.

Cheng et al. studied the effect of different substances on mortality and other clinical outcomes, such as operative interventions, hospital stay, and ICU stays. Their study showed that benzodiazepine positive was not associated with mortality, whereas the concurrent use of benzodiazepine with opioids had higher odds of mortality (Cheng et al. 2016). When used alone, benzodiazepine was associated with increased mechanical ventilation and ICU admission. Similarly, patients positive for opioids only have lower odds of ICU admission and mechanical ventilation, which supports the result in our study that shows increased HRU among patients using opioids and benzodiazepine combinations (Cheng et al. 2016).

Given the limited resources available in trauma centers specifically the mechanical ventilation and ICU, it is essential to consider the efficient resource allocation strategies. Right treatment and health care services can improve the patient’s outcomes and reduce the economic burden to both payers and heath care systems. The result of the study enhances the right decision and right treatment to right patient. Overall, the result of the study is significant in optimizing the health care delivery and patient’s outcome.

While previous studies have shown the effect of each drug separately on mortality and other outcomes among trauma patients, this study is the first to systematically describe the effect of opioid-related polysubstance use based on the number and types of combinations on mortality and HRU. Moreover, this study is the first to conduct polysubstance research using a national database, which increases its generalizability. This study also provides unique evidence on the effect of opioid-related pre-injury polysubstance use on mortality and HRU among trauma patients. The baseline for the study was established with the opioid-only group. Comparing the effect of polysubstance use with opioids only provides evidence of how different combinations affect mortality and HRU. Our study results will further inform healthcare providers and policymakers about the challenges posed by polysubstance use in trauma patients and target population to tailor their intervention to prevent polysubstance use. Additionally, the combination of substances is of greater importance because different combinations of drugs have different effects on patients and health outcomes. Hence, considering polysubstance use while treating patients in trauma centers will help to improve health outcomes.

There are four notable limitations to our study. First, because the NTDB lacks information on the number of substances seen in the blood, the data about the drug screening was limited to the drug’s presence. Therefore, we could not incorporate the effect of the blood concentration of drugs on outcomes. Second, the database did not provide whether positively tested drugs were prescribed. For example, if the patients were transported by ambulance or transferred from another hospital, the database did not show if they were administered narcotics or benzodiazepine as a portion of their treatment. Our study did not incorporate the possibility of the drug being prescribed for clinical purposes, which is a major limitation. The third limitation is related to the database itself. NTDB is a national database and is limited by voluntary participation from the trauma centers. Only 29.4% have the substance screening performed and hence, the results are prone to testing bias. Similarly reporting bias and coding errors can then be a potential restriction in using this database. Finally, our study did not incorporate socioeconomic factors except for the patient’s age, sex, and race. Other socioeconomic factors like education, marital status, and income could affect the study’s outcomes.

## Conclusion

Opioid-related pre-injury polysubstance use was not associated with mortality. Nevertheless, the use of opioids and benzodiazepine is associated with an overall increase in HRU outcomes (i.e., need for hospital admission, ICU, and mechanical ventilation.) Polysubstance use is a significant problem among trauma patients, which bears large economic burdens from increased health services utilization. Future work is required to further explore the interaction between different substances and their effects on patients, other health outcomes and health services utilization to understand the effect of pre-injury polysubstance use among trauma patients.

### Supplementary Information

**Additional file 1**. Table S1, Table S2.

## Data Availability

The data that support the findings of this study are available from American Colleges of Surgeons (ACS), but restrictions apply to the availability of these data, which were used under license for the current study, and so are not publicly available. Data are however available from authors upon reasonable request and with permission of ACS.

## Abbreviations

HRU: Health Resources Utilization
NTDB: National Trauma Databank
ACS: American College of Surgeons
BAC: Blood Alcohol Concentration
ICU: Intensive Care Unit
AUD: Alcohol Use Disorder
SUD: Substance Use Disorder
CHF: Congestive Heart Failure
CRD: Chronic Renal Disease
COPD: Chronic Obstructive Pulmonary Disease
SBP: Systolic Blood Pressure
GCS: Glasgow Coma Scale
OR: Odds ratio
IQR: Interquartile Range

## References

[CR1] National Institute on Drug Abuse (NIDA). Overdose Death Rates [Internet]. [cited 2022 Aug 4]. Available from: https://nida.nih.gov/research-topics/trends-statistics/overdose-death-rates.

[CR2] Andelic N, Jerstad T, Sigurdardottir S, Schanke A-K, Sandvik L, Roe C (2010). Effects of acute substance use and pre-injury substance abuse on traumatic brain injury severity in adults admitted to a trauma centre. J Trauma Manag Outcomes.

[CR3] Barocas JA, Wang J, Marshall BDL, LaRochelle MR, Bettano A, Bernson D (2019). Sociodemographic factors and social determinants associated with toxicology confirmed polysubstance opioid-related deaths. Drug Alcohol Depend.

[CR4] Cannon R, Bozeman M, Miller KR, Smith JW, Harbrecht B, Franklin G (2014). The prevalence and impact of prescription controlled substance use among injured patients at a Level I trauma center. J Trauma Acute Care Surg.

[CR5] Cheng V, Inaba K, Johnson ME, Byerly SE, Jiang Y, Matsushima K (2016). The impact of pre-injury controlled substance use on clinical outcomes after trauma. J Trauma Acute Care Surg.

[CR6] Community Impact North Carolina (CINC). Polysubstance use among people who use opioids or other drugs nonmedically. 2020.

[CR7] Compton WM, Valentino RJ, DuPont RL (2021). Polysubstance use in the U.S. opioid crisis. Mol Psychiatry.

[CR8] Cornwell EE, Belzberg H, Velmahos G, Chan LS, Demetriades D, Stewart BM (1998). The prevalence and effect of alcohol and drug abuse on cohort-matched critically injured patients. Am Surg.

[CR9] Cowperthwaite MC, Burnett MG (2011). Treatment course and outcomes following drug and alcohol-related traumatic injuries. J Trauma Manag Outcomes.

[CR10] Culhane J, Freeman C (2020). The effect of illegal drug screening results and chronic drug use on perioperative complications in trauma. J Emerg Trauma Shock.

[CR11] Demetriades D, Gkiokas G, Velmahos GC, Brown C, Murray J, Noguchi T (2004). Alcohol and illicit drugs in traumatic deaths: prevalence and association with type and severity of injuries. J Am Coll Surg.

[CR12] Dubois S, Mullen N, Weaver B, Bédard M (2015). The combined effects of alcohol and cannabis on driving: impact on crash risk. Forensic Sci Int.

[CR13] Gibbons RD, Hur K, Quinn PD (2021). Concomitant opioid and benzodiazepine use and risk of suicide attempt and intentional self-harm: pharmacoepidemiologic study. Drug Alcohol Depend.

[CR14] Gladden RM, O’Donnell J, Mattson CL, Seth P (2019). Changes in opioid-involved overdose deaths by opioid type and presence of benzodiazepines, cocaine, and methamphetamine—25 States, July-December 2017 to January-June 2018. MMWR Morb Mortal Wkly Rep.

[CR15] Hadjizacharia P, Green DJ, Plurad D, Chan LS, Inaba K, Shulman I (2009). Methamphetamines in trauma: effect on injury patterns and outcome. J Trauma Acute Care Surg.

[CR16] Hashmi ZG, Kaji AH, Nathens AB (2018). Practical guide to surgical data sets: National Trauma Data Bank (NTDB). JAMA Surg.

[CR17] Hassan AN, Le Foll B (2019). Polydrug use disorders in individuals with opioid use disorder. Drug Alcohol Depend.

[CR18] Hernandez I, He M, Brooks MM, Zhang Y (2018). Exposure-response association between concurrent opioid and benzodiazepine use and risk of opioid-related overdose in Medicare Part D beneficiaries. JAMA Netw Open.

[CR19] Karnick AT, Caulfield NM, Bauer BW, Martin RL, Kaufman EJ, Winchell R (2021). Substance use and suicide outcomes among self-injured trauma patients. Drug Alcohol Depend.

[CR20] Korthuis PT, Cook RR, Foot CA, Leichtling G, Tsui JI, Stopka TJ (2022). Association of methamphetamine and opioid use with nonfatal overdose in rural communities. JAMA Netw Open.

[CR21] Neeki MM, Dong F, Liang L, Toy J, Carrico B, Jabourian N (2018). Evaluation of the effect of methamphetamine on traumatic injury complications and outcomes. Addict Sci Clin Pract.

[CR22] Pandya U, O’Mara MS, Wilson W, Opalek J, Lieber M (2015). Impact of preexisting opioid use on injury mechanism, type, and outcome. J Surg Res.

[CR23] Park TW, Saitz R, Ganoczy D, Ilgen MA, Bohnert ASB (2015). Benzodiazepine prescribing patterns and deaths from drug overdose among US veterans receiving opioid analgesics: case-cohort study. BMJ.

[CR24] Bureau of Justice Assistance (BJA.gov). Polysubstance Use Among People Who Use Opioids. Comprehensive Opioid, Stimulant, and Substance Use Program. 2018; 1–5.

[CR25] Satish S, Freeman C, Culhane J (2021). Urine drug screen positive for cocaine and amphetamine is not an adverse risk factor for cardiovascular morbidity or mortality in trauma. Trauma Surg Acute Care Open.

[CR26] Silver CM, Visenio MR, Thomas AC, Reddy S, Raven MC, Kanzaria HK (2023). Hospital variability in adoption of alcohol and drug screening in adult trauma patients. J Trauma Acute Care Surg.

[CR27] Ungar WJ, Coyte PC (1998). Health services utilization reporting in respiratory patients. J Clin Epidemiol.

[CR28] White JM, Irvine RJ (1999). Mechanisms of fatal opioid overdose. Addiction.

[CR29] Witkiewitz K, Vowles KE (2018). Alcohol and opioid use, co-use, and chronic pain in the context of the opioid epidemic: a critical review. Alcohol Clin Exp Res.

[CR30] Yang BR, Oh I-S, Li J, Jeon H-L, Shin J-Y (2020). Association between opioid analgesic plus benzodiazepine use and death: a case-crossover study. J Psychosom Res.

[CR31] Yeung JT, Williams J, Bowling WM (2013). Effect of cocaine use on outcomes in traumatic brain injury. J Emerg Trauma Shock.

[CR32] Zhang Z (2016). Multiple imputation with multivariate imputation by chained equation (MICE) package. Ann Transl Med.

