# Supplementary material for: Opioid-related polysubstance use and its effect on mortality and health resource utilization among trauma patients
Source: Inj Epidemiol. 2023 Oct 23;10:54. doi: 10.1186/s40621-023-00459-0 (PMC10594664; doi:10.1186/s40621-023-00459-0)
Supplement: Supplementary file 1 — Additional file 1. Table S1, Table S2. [file 40621_2023_459_MOESM1_ESM.docx]

Table S1: Demographics of trauma patients with pre-injury polysubstance by type of substances

|  | **Opioids(n=17930)** | **Opioids and alcohol(3740)** | **Opioids and Benzodiazepines (n=4076)** | **Opioids and Stimulants (n=6126)** | **Opioids, stimulants and Benzodiazepines (n=1611)** | **Opioids alcohol and benzodiazepines (n=895)** |
| --- | --- | --- | --- | --- | --- | --- |
| **Age yrs, n(%)** |  |  |  |  |  |  |
| ≤21 | 736(60.83) | 122(10.08) | 167(13.80) | 130(10.74) | 36(2.98) | 19(1.57) |
| 22-34 | 3216(41.80) | 949(12.34) | 812(10.56) | 1953(25.39) | 542(7.05) | 221(2.87) |
| 35-44 | 2238(39.72) | 652(11.57) | 627(11.13) | 1536(27.26) | 426(7.56) | 156(2.77) |
| 45-64 | 5932(48.75) | 1546(12.71) | 1516(12.46) | 2212(18.18) | 538(4.42) | 424(3.48) |
| ≥65 | 5808(75.70) | 471(6.14) | 954(12.43) | 295(3.85) | 69(0.90) | 75(0.98) |
| **Sex, n (%)** |  |  |  |  |  |  |
| Female | 6903(57.10) | 979(8.10) | 1658(13.71) | 1759(14.55) | 544(4.50) | 247(2.04) |
| Male | 11026(49.47) | 2761(12.39) | 2418(10.85) | 4367(19.59) | 1067(4.79) | 648(2.91) |
| **Race/Ethnicity, n (%)** |  |  |  |  |  |  |
| White | 13587(52.75) | 2642(10.26) | 3268(12.69) | 4352(16.90) | 1243(4.83) | 666(2.59) |
| African American | 2214(47.53) | 598(12.84) | 432(9.27) | 1067(22.91) | 215(4.62) | 132(2.83) |
| Asian | 362(71.97) | 32(6.36) | 40(7.95) | 52(10.34) | 9(1.79) | 8(1.59) |
| American indian | 120(37.50) | 86(26.88) | 23(7.19) | 65(20.31) | 7(2.19) | 19(5.94) |
| Pacific Islander | 21(38.89) | 12(22.22) | 9(16.67) | 10(18.52) | 2(3.70) | 0(0) |
| Other | 1544(53.06) | 350(12.03) | 284(9.76) | 536(18.42) | 129(4.43) | 67(2.30) |
| Missing | 82(46.86) | 20(11.43) | 20(11.43) | 44(25.14) | 6(3.43) | 3(1.71) |
| **Mechanism Category, n (%)** |  |  |  |  |  |  |
| Auto vs Pedesterian | 632(41.94) | 180(11.94) | 128(8.49) | 444(29.46) | 80(5.31) | 43(2.85) |
| Fall | 6811(61.72) | 1178(10.67) | 1412(12.79) | 1097(9.94) | 279(2.53) | 259(2.35) |
| Motorcycle collision | 1232(55.87) | 235(10.66) | 229(10.39) | 381(17.28) | 76(3.45) | 52(2.36) |
| Motor vehicle collision | 5417(51.38) | 1123(10.65) | 1325(12.57) | 1834(17.39) | 612(5.80) | 233(2.21) |
| Penetrating | 1227(35.50) | 371(10.73) | 482(13.95) | 949(27.46) | 272(7.87) | 155(4.48) |
| Other | 2401(46.51) | 590(11.43) | 448(8.68) | 1311(25.40) | 274(5.31) | 138(2.67) |
| Missing | 210(44.87) | 63(13.46) | 52(11.11) | 110(23.50) | 18(3.85) | 15(3.21) |
| **Payment, n (%)** |  |  |  |  |  |  |
| Public | 8235(51.31) | 1559(9.71) | 1954(12.18) | 3125(19.47) | 782(4.87) | 394(2.45) |
| Private | 6924(56.89) | 1400(11.50) | 1520(12.49) | 1542(12.67) | 469(3.85) | 315(2.59) |
| Uninsured | 1664(40.10) | 580(13.98) | 370(8.92) | 1101(26.53) | 292(7.04) | 143(3.45) |
| Other | 954(57.68) | 147(8.89) | 192(11.61) | 271(16.38) | 54 0.16 3.26 3.35 | 36(2.18) |
| Missing | 153(43.10) | 54(15.21) | 40(11.27) | 87(24.51) | 14(3.94) | 7(1.97) |
| **Comorbidities n (%)** |  |  |  |  |  |  |
| Alcohol Use Disorder | 824(28.93) | 993(34.87) | 269(9.45) | 394(13.83) | 94(3.30) | 274(9.62) |
| Substance Use Disorder | 1174(22.67) | 394(7.61) | 516(9.97) | 2329(44.98) | 630(12.17) | 135(2.61) |
| Chronic Heart Failure | 744(68.38) | 67(6.16) | 155(14.25) | 80(7.35) | 21(1.93) | 21(1.93) |
| Chronic Renal Disease | 208(70.27) | 16(5.41) | 43(14.53) | 23(7.77) | 3(1.01) | 3(1.01) |
| Smoker | 3745(36.63) | 1350(13.21) | 1176(11.50) | 2881(28.18) | 746(7.30) | 325(3.18) |
| Hypertension | 6639(62.77) | 1031(9.75) | 1295(12.24) | 1062(10.04) | 275(2.60) | 275(2.60) |
| COPD | 16418(51.72) | 3491(11.00) | 3660(11.53) | 5822(18.34) | 1521(4.79) | 834(2.63) |
| Diabetes | 2788(66.70) | 292(6.99) | 512(12.25) | 424(10.14) | 102(2.44) | 62(1.48) |
| **Injury Severity, n (%)** |  |  |  |  |  |  |
| Less severe | 14711(53.16) | 3016(10.90) | 3163(11.43) | 4896(17.69) | 1207(4.36) | 681(2.46) |
| Severe | 3160(47.89) | 711(10.77) | 900(13.64) | 1217(18.44) | 399(6.05) | 212(3.21) |
| Missing | 59(56.19) | 13(12.38) | 13(12.38) | 13(12.38) | 5(4.76) | 2(1.90) |
| GCS, median | 15 | 15 | 15 | 15 | 15 | 15 |
| Pulse Rate, median | 87 | 90 | 90 | 92 | 95 | 93 |
| SBP, median mmHG | 140 | 131 | 134 | 135 | 130 | 128 |

Abbreviation: ISS- Injury Severity Score, COPD- Chronic obstructive pulmonary disease, GCS- Glasgow Coma Scale and SBP- Systolic Blood Pressure

|  | |  |
| --- | --- | --- |
| **Risk Factors** | **OR^a^ (95% CI)** | **p-value** |
| **Polysubstance by number of substances** | | |
| No Substance | **Reference** |  |
| Opioids only | 0.86(0.75-0.99) | 0.04 |
| Opioids+1 substance | 0.83(0.72-0.95) | 0.006 |
| Opioids+≥2 substance | 0.73 (0.64-0.83) | <0.0001 |
| **Polysubstance by types of substances** | | |
| No Substance | **Reference** |  |
| Opioids only | 0.86(0.75-0.99) | 0.04 |
| Opioids and alcohol | 0.77(0.56-1.06) | 0.11 |
| Opioids and stimulants | 0.98(0.77-1.25) | 0.9 |
| Opioids and benzodiazepines | 0.72(0.56-0.92) | 0.008 |
| Opioids, stimulants and benzodiazepines | 0.60(0.37-0.96) | 0.03 |
| Opioids, alcohol and benzodiazepines | 0.44(0.22-0.87) | 0.02 |

Table S2- Odds of mortality for the no substance as comparison group

^a^ Logistic regression model was adjusted for age, sex, mechanism of injury, injury severity, Glasgow Coma Scale and systolic blood pressure, pulse rate and different comorbid conditions.

|  | **Hospital Admission** | | **ICU Admission** | | **Mechanical Ventilation** | |
| --- | --- | --- | --- | --- | --- | --- |
|  | **Odds ratio ^a^, CI** | **p-value** | **Odds ratio ^a^, CI** | **p-value** | **Odds ratio ^a^, CI** | **p-value** |
| **Polysubstance use by number of substances** | | | | | | |
| No substance | **Reference** |  | **Reference** |  |  |  |
| Opioids only | 1.2(1.16-1.24) | <0.0001 | 0.91(0.88-0.95) | <0.0001 | 0.60(0.56-0.64) | <0.0001 |
| Opioids+1 substance | 1.1(1.12-1.19) | <0.0001 | 0.96(0.93-1.001) | 0.054 | 0.79(0.74-0.83) | <0.0001 |
| Opioids+≥2 substance | 1.3(1.34-1.43) | <0.0001 | 0.95(0.91-0.98) | 0.005 | 0.91(0.86-0.96) | 0.001 |
| **Polysubstance Use by type of substances** | | | | | | |
| No substance | **Reference** |  | **Reference** |  | **Reference** |  |
| Opioids Only | 1.2(1.16-1.24) | <0.0001 | 0.91(0.88-0.95) | <0.0001 | 0.60(0.56-0.64) | <0.0001 |
| Opioids and alcohol combination | 1.13(1.05-1.21) | 0.0005 | 0.84(0.77-0.91) | <0.0001 | 0.65(0.57-0.75) | <0.0001 |
| Opioids and stimulants | 1.07(1.02-1.13) | 0.01 | 1.01(0.95-1.07) | 0.69 | 0.85(0.77-0.94) | 0.0021 |
| Opioids and benzodiazepines | 1.36(1.27-1.45) | <0.0001 | 1.14(1.06-1.23) | 0.0003 | 1.03(0.92-1.15) | 0.58 |
| Opioids, stimulants and benzodiazepines | 1.12(1.01-1.25) | 0.03 | 1.34(1.19-1.51) | <0.0001 | 1.52(1.30-1.77) | <0.0001 |
| Opioids, alcohol and benzodiazepines | 1.38(1.19-1.60) | <0.0001 | 1.23(1.05-1.43) | 0.008 | 0.97(0.78-1.22) | 0.85 |

Table S3 : Odds of Health Resource Utilization for the no substance as comparison group

^a^ Logistic regression model was adjusted for age, sex, mechanism of injury, injury severity, Glasgow Coma Scale and systolic blood pressure, pulse rate and different comorbid conditions.
